# High Expression of Fas-Associated Factor 1 Indicates a Poor Prognosis in Non-Small-Cell Lung Cancer

**DOI:** 10.3390/curroncol30110687

**Published:** 2023-10-25

**Authors:** De Hu, Hidetaka Yamada, Katsuhiro Yoshimura, Tsutomu Ohta, Kazuo Tsuchiya, Yusuke Inoue, Kazuhito Funai, Takafumi Suda, Yuji Iwashita, Takuya Watanabe, Hiroshi Ogawa, Nobuhito Kurono, Kazuya Shinmura, Haruhiko Sugimura

**Affiliations:** 1 Department of Tumor Pathology, Hamamatsu University School of Medicine, Hamamatsu 431-3192, Shizuoka, Japan; d18035kk@hama-med.ac.jp (D.H.); kyoshimura@mdanderson.org (K.Y.); tohta@hm.tokoha-u.ac.jp (T.O.); k179825@siz.saiseikai.or.jp (K.T.); yinoue@hama-med.ac.jp (Y.I.); 07485223@hama-med.ac.jp (Y.I.); kzshinmu@hama-med.ac.jp (K.S.); 2 Second Division, Department of Internal Medicine, Hamamatsu University School of Medicine, Hamamatsu 431-3192, Shizuoka, Japan; suda@hama-med.ac.jp; 3 Department of Physical Therapy, Faculty of Health and Medical Sciences, Tokoha University, Hamamatsu 431-2102, Shizuoka, Japan; 4 First Department of Surgery, Hamamatsu University School of Medicine, Hamamatsu 431-3192, Shizuoka, Japan; kfunai@hama-med.ac.jp; 5 Division of Thoracic Surgery, Department of Respiratory Disease Center, Seirei Mikatahara General Hospital, Hamamatsu 433-8558, Shizuoka, Japan; takuya.watanabe@sis.seirei.or.jp; 6 Department of Pathology, Seirei Mikatahara General Hospital, Hamamatsu 433-8558, Shizuoka, Japan; h-ogawa@sis.seirei.or.jp; 7 Department of Chemistry, Hamamatsu University School of Medicine, Hamamatsu 431-3192, Shizuoka, Japan; chrono@hama-med.ac.jp; 8 Sasaki Institute, Sasaki Foundation, Tokyo 101-0062, Japan

**Keywords:** FAF1, non-small-cell lung cancer, prognosis, apoptosis, proliferation

## Abstract

Fas-associated factor 1 (FAF1) is a death-promoting protein identified as an interaction partner of the death receptor Fas. The downregulation and mutation of FAF1 have been reported in a variety of human tumors, but there have been few studies on lung cancer. Here, we investigated the prognostic significance of FAF1 expression in non-small-cell lung cancer (NSCLC), and whether aberrant FAF1 expression may be involved in the pathogenesis and prognosis of NSCLC. FAF1 expression was examined in NSCLC specimens as well as human lung cancer cell lines. In addition, changes in cell viability and apoptosis upon regulating FAF1 expression were investigated in lung cancer cell lines. As a result, high FAF1 expression was significantly associated with a poor prognosis in NSCLC. In lung cancer cell lines, FAF1 downregulation hindered cell viability and tended to promote early apoptosis. In conclusion, this is the first study of the clinical significance of FAF1 in NSCLC, showing that FAF1 overexpression is associated with a poor prognosis in NSCLC and that FAF1 acts as a dangerous factor rather than an apoptosis promoter in NSCLC.

## 1. Introduction

Lung cancer remains the most commonly diagnosed cancer and the leading cause of cancer death worldwide according to the GLOBOCAN 2018 database [1]. The development of molecular markers in various subtypes of lung cancer has been one of the most significant advances in “Precision Medicine” in the last decade [2,3]. However, much remains to be learned about the process of lung cancer, particularly non-small-cell lung cancer (NSCLC).

The protein encoded by the Fas-associated factor 1 (FAF1) gene binds to the Fas antigen and is a member of Fas-death-inducing signaling complex, acting upstream of caspase-8, and potentiates but does not initiate apoptosis [4,5,6]. FAF1 is a scaffold protein containing a FAS-interacting domain (FID) and a death effector domain-interacting domain (DEDID), and also a ubiquitin receptor containing the ubiquitin-associating (UBA), ubiquitin-like 1 and 2 (UBL1, UBL2) and ubiquitin regulatory X (UBX) domains [7,8]. It has been proposed that it functions as a tumor suppressor because it participates in several processes that promote cell death [9,10,11,12,13].

FAF1 is located at chromosome 1p32.3, where a loss of heterozygosity occurs in many cancers [14]. FAF1 deficiency has been linked to uterine cervical carcinoma, mantle cell lymphoma, and malignant mesothelioma [13,15,16]. The downregulation of FAF1 mRNA has also been reported in gastric cancer and lung cancer [17,18,19]. A somatic FAF1 mutation was discovered in a case of recurrent sarcoma, and germline mutations of FAF1 were recently discovered in hereditary colorectal cancer [20,21,22]. Aberrant FAF1 is linked to the development of various cancers according to previous reports, but the link is not clear. Ryu et al. investigated the tissue distribution of FAF1, and discovered that FAF1 mRNA is scarcely expressed in the lung, placenta, brain, liver, spleen, and kidney, whereas, FAF1 protein is expressed in a variety of human cancer cell lines [5]. 

Furthermore, it has been reported that FAF1’s functions and cellular locations are sometimes inconsistent. FAF1 is localized not only in the cytoplasm where it interacts with Fas, but also in the nucleus. It modulates a variety of biological processes other than cell death, by interacting with diverse molecules. FAF1 contains phosphorylation sites for protein kinase casein kinase 2 (CK2) within the nuclear targeting domain, and it also inhibits the nuclear factor kappa-light-chain-enhancer of activated B cells (NF-κB) by interfering with the nuclear translocation of the p65 subunit. Furthermore, it interacts with valosin-containing protein (VCP), which is involved in the ubiquitin–proteasome pathway. FAF1 translocates into the nucleus in response to oxidative stress, where it binds to Poly(ADP-Ribose) Polymerase 1 (PARP1) to positively regulate the enzymatic activity of PARP1 [5,9,23,24,25,26]. In this regard, FAF1 may be complicatedly regulated and may play different roles depending on the tissue-specific context.

The goal of this study was to gain histopathological insight into the potential role of FAF1 in NSCLC. We investigated FAF1 protein expression in NSCLC and how it affects lung cancer cell viability and apoptosis progression in vitro.

## 2. Materials and Methods

### 2.1. Specimen Preparation

We collected 609 resected NSCLC tumor specimens, including 397 and 212 patients who had received curative surgical treatment between January 1990 and December 2013 at Hamamatsu University Hospital and between January 2006 and April 2014 at Seirei Mikatahara General Hospital, respectively. All patients provided informed consent for us to use these specimens for medical research. This study was authorized by the Institutional Review Board of Hamamatsu University School of Medicine (IRB 20-011) and Seirei Mikatahara General Hospital (IRB 20-36). This research adheres to the Helsinki Declaration and Japanese regulations [27]. Clinical and pathological data, such as age, gender, tumor stage, smoking history, and outcomes, were retrospectively obtained from the patients’ medical records. Pathologically confirmed operative cases were included, while histopathological confirmation of viable tissue was not available. Cases that had pathological evidence of lung cancer and available tissue for pathological characterization were included, and cases in which a conclusive pathological diagnosis was not available or the tissue was too small to characterize for further analysis were excluded. All of the specimens were fixed with formalin, embedded in paraffin, and punched out of distinct tumor areas with 3 mm diameter cylinders by senior pathologists. Tissue microarray (TMA) cores were isolated from representative lung cancer tissues and their corresponding normal lung tissues. The presence of cells in TMA cores was validated by reviewing hematoxylin- and eosin-stained sections, as reported previously [28,29]. Tumors were classified into pathological stages I–III according to the 8th edition of the TNM classification and histologically classified by three senior pathologists (KY, HO, HS) based on the 2015 *World Health Organization Classification of Tumors of the Lung*, *Pleura*, *Thymus*, *and Heart* and the 2021 World Health Organization Classification of Tumors of the Pleura: Advances since the 2015 Classification [30].

### 2.2. Immunohistochemistry (IHC) Analysis

TMA sections were subjected to a standard IHC protocol. Briefly, formalin-fixed paraffin-embedded (FFPE) sections were deparaffinized and rehydrated. Endogenous peroxidase was inhibited with hydrogen peroxide after antigen retrieval [31]. The cells were then incubated with primary antibodies overnight, before being incubated with secondary antibodies. This study made use of FAF1 (EPR14754, Abcam, Cambridge, UK), EGFR E746-A750 deletion-specific (D6B6, Cell Signaling Technology, Danvers, MA, USA), and EGFR L858R mutant-specific (43B2, Cell Signaling Technology) antibodies, and the secondary antibody used was N-Histofine Simple Stain MAX-PO MULTI (724152; Nichirei Biosciences). The expression levels of FAF1 protein were graded by three independent individuals (DH, HY, and HS) as follows: 0, no staining or faint staining of tumor cells <10%; 1, faint staining of tumor cells >10%; 2, moderate staining; and 3, strong staining. Grades 1–3 were assigned to positive expression, while grade 0 was assigned to negative expression. In the cases where the first estimation process produced an inconsistent grade, three cases were discussed and a final grade was assigned. EGFR mutant statuses were assessed using a previous protocol [32]. Specimens with no or faint staining in <10% of tumor cells were classified as negative, while the rest were classified as positive. All slides were examined using a bright-field microscope (Leica DMD 108; Leica Microsystems, Wetzlar, Germany).

### 2.3. Survival Analysis Using Kaplan–Meier Plotter

The overall survival analysis of NSCLC at the FAF1 mRNA expression levels was performed using the Kaplan–Meier plotter (https://kmplot.com/analysis/index.php?p=service&cancer=lung (accessed on 30 August 2023)). As recommended at the site, FAF1 (224217_s_at) was chosen, and low and high expressions were split by median expression. The follow-up threshold was assigned as 10 years because the curves crossed over afterwards and FAF1 expression became no longer relevant after 10 years. Nine cohorts (GSE157011, GSE102287, GSE19188, GSE29013, GSE30219, GSE31210, GSE3141, GSE37745, GSE50081) of 1411 cases from the database were employed for prognostic analysis.

### 2.4. TUNEL Assay

To identify apoptotic cells in NSCLC, DNA fragmentation was carried out via the TUNEL method using the In Situ Apoptosis Detection Kit (Takara Biomedicals, Kusatsu, Japan) according to the manufacturer’s instructions. Briefly, FFPE sections were deparaffinized and pretreated with proteinase K for 15 min at room temperature. After blocking endogenous peroxidase activity with 3% H_2_O_2_ for 5 min, sections were incubated with terminal deoxynucleotidyl transferase for 60 min at 37 °C. After washing with PBS, sections were incubated with anti-FITC-HRP conjugate for 30 min at 37 °C, followed by color development with DAB•H_2_O_2_ for 10 min at room temperature and 3% methyl green solution. The presence of nuclear staining was interpreted as TUNEL-positive cells, compared to the TUNEL-negative cells, which were not stained at all. The proportion of it was estimated from the number of positive cells. Observation and image capture were conducted using a NanoZoomer 2.0-HT Digital Pathology System (Hamamatsu Photonics, K.K., Hamamatsu, Japan).

### 2.5. Cell Culture

Human NSCLC cell lines A549, H460, PC9, and H1299, as well as the normal human bronchial epithelial cell line NHBE, were acquired from the American Type Culture Collection (ATCC, Manassas, MD, USA). Cells were cultured in RPMI-1640 medium (Sigma-Aldrich, St. Louis, MO, USA) supplemented with 10% fetal bovine serum and 1% penicillin-streptomycin. Cells were kept in a humidified atmosphere with 5% CO_2_ at 37 °C. 

### 2.6. Small Interfering RNA (siRNA) Transfection

A Silencer Select Pre-Designed siRNA for FAF1 (Cat #4392420, s21934 and s21935, Invitrogen, Carlsbad, CA, USA) and Silencer Select Negative Control (Cat #4390843, Invitrogen, Carlsbad, CA, USA) were acquired for the knockdown assay. The sequences were as follows: FAF1-siRNA1, 5′-GGCAGAUUGUAGAAAGGCAtt-3′; FAF1-siRNA2, 5′-CACCGAUGUUCAUAUGGUUtt-3′. The lung cancer cell line A549 was cultured for 24 h before transfection and transfected with final concentrations of 15 nM siRNA using Opti-MEM (Gibco, Waltham, MA, USA) and lipofectamine 2000 (Thermo Fisher Scientific, Waltham, MA, USA) in RPMI-1640 medium (Sigma-Aldrich, St. Louis, MO, USA) supplemented with 10% fetal bovine serum. The cells were used for further assays after 48 h. The efficiency of siRNA transfection was evaluated via Western blotting.

### 2.7. Plasmid Construction and Transfection

Total RNA was extracted from the gastric surgical specimen from Hamamatsu University Hospital (IRB 20-011) using the RNeasy Mini Kit (Qiagen, Valencia, CA, USA) as directed by the manufacturer. Total RNA was reverse-transcribed using first-strand cDNAs using the SuperScript First-Strand Synthesis System for RT-PCR (Thermo Fisher Scientific) according to the manufacturer’s instructions. Full-length cDNA of hFAF1 was generated via PCR using stomach cDNA as a template with sense (5′-ATATATGAACGAATTCATGGCGTCCAACATGGACCGGGAGATGATCCTGGCGGATTTTCAG-3′) and antisense (5′-GGATAATTGGCTCGAGTTACTCTTTTGCTTCAAGGAAAAGGGTTTCTTGA-3′) primers containing EcoRI and XhoI linkers, respectively. Primers were designed based on FAF1 transcripts (Accession No. NM_007051), using sequences that have low similarity to other genes upon searching using the Basic Local Alignment Search Tool (BLAST) of the National institutes of Health (NIH) (https://blast.ncbi.nlm.nih.gov/Blast.cgi (accessed on 2 October 2020)). The PCR-amplified fragment was ligated into the EcoRI/XhoI site of the pCMV-tag2C (Promega), which contained 3xFlag-His6 tags in the N-terminus. The cDNA of hFAF1 was validated via DNA sequencing.

The lung cancer cell line H460 was transfected with an expression plasmid vector using Opti-MEM (Gibco, Waltham, MA, USA) and Lipofectamine 3000 (Thermo Fisher Scientific) according to the manufacturer’s instructions. After 48 h, the efficiency of overexpression was determined using immunofluorescence analysis, and the cells were used in subsequent experiments.

### 2.8. Western Blot Analysis

After washing with phosphate-buffered saline (PBS), cells were lysed with 1× SDS sample buffer (69.4 mM Tris-HCL pH 6.8, 2.8% SDS, 11.1% glycerol) [33], followed by sonication treatment. The protein concentration was determined using the Pierce BCA Protein Assay Kit (Thermo Fisher Scientific). A total of 10 µg denatured protein samples were separated using SDS-PAGE, and then, transferred to PVDF membranes (Cytiva, Chicago, IL, USA) using a Trans-Blot Turbo Cassette (Bio-Rad, Hercules, CA, USA). The membranes were blocked with 5% skim milk and incubated with rabbit anti-FAF1 monoclonal antibody (EPR14754, Abcam), mouse anti-GAPDH monoclonal antibody (6C5, Abcam), or rabbit anti-Flag polyclonal antibody (Sigma-Aldrich, St. Louis, MO, USA) overnight at 4 °C, followed by incubation with anti-rabbit (NA9340, Cytiva) or anti-mouse (NA9310, Cytiva) secondary antibody at room temperature for 1 h. Western blots were prepared using Pierce ECL Plus Western blotting substrate (Thermo Fisher Pierce, Rockford, IL, USA), and then, exposed to the Fusion FX7 system (Vilber Lourmat, Marne-la-Vallée, France). Protein expression was quantitated using FUSION FX7 software. The results were normalized using GAPDH as an internal control.

### 2.9. RNA Isolation and Quantitative Real-Time PCR (qPCR)

Total RNA was isolated using the RNeasy Plus Mini Kit (Qiagen, Valencia, CA, USA) as directed by the manufacturer. Total RNA was reverse-transcribed using ReverTra Ace qPCR RT Master Mix (Toyobo, Osaka, Japan) according to the manufacturer’s instructions. qPCRs were carried out in triplicate using a StepOne Plus Real-Time PCR System instrument (Thermo Fisher Scientific) using a TaqMan qPCR Assay (Thermo Fisher Scientific). FAF1 Hs01568310_m1 (FAM-MGB) (Cat #4351372, Thermo Fisher Scientific), and GAPDH Hs03929097_g1 (VIC-MGB) (Cat #4448484, Thermo Fisher Scientific) were used with primers and probe sets optimized by the manufacturer (https://www.thermofisher.cn/taqman-gene-expression/product/Hs01568310_m1?CID=&ICID=&subtype= (accessed on 2 July 2020)), and the primer sequences were unrevealed. The qPCR reaction was performed under the following conditions: polymerase activation at 95 °C for 10 min, 40 cycles of denaturation at 95 °C for 15 sec, and annealing/extension at 60 °C for 1 min. Target gene expression levels were normalized to the level of GAPDH. The relative expression was examined using the ∆∆Ct method.

### 2.10. Immunofluorescence Analysis

Cells were fixed with 4% paraformaldehyde, permeabilized with methanol, and incubated for 1 h at room temperature with rabbit anti-Flag polyclonal antibody (Sigma-Aldrich, St. Louis, MO, USA), followed by incubation for 1 h at room temperature with Alexa Fluor 488 goat anti-rabbit IgG (H + L) Cross-Adsorbed Secondary Antibody (Thermo Fisher Scientific). Nuclei were stained with 4′,6-diamidino-2-phenylindole (DAPI). Slides were imaged using a fluorescence microscope (Olympus IX83, Tokyo, Japan). The proportion of fluorescence-positive cells was calculated in seven randomly selected fields.

### 2.11. Cell Viability Assay

Cells were seeded in 96-well plates at 3000 cells per well after 48 h of siRNA transfection. The viability and proliferation abilities of cells were determined using a Cell Counting Kit-8 (CCK-8; Dojindo, Kumamoto, Japan) according to the manufacturer’s protocol. Cells were incubated with 10% CCK-8 for 1.5 h, and the absorbance at 450 nm was measured in each well via spectrophotometry (Synergy HT, BioTek) every 24 h [32,34]. 

### 2.12. Apoptosis Analysis

An annexin V-FITC Apoptosis Detection Kit (Nacalai Tesque, Kyoto, Japan) was used to identify apoptosis by measuring annexin V and propidium iodide-positive cells according to the manufacturer’s protocol. Briefly, cells were collected and washed with PBS, before being incubated with 5 mL of annexin V-FITC solution and 5 mL of propidium iodide solution for 15 min at room temperature. Thereafter, apoptosis levels were assessed using flow cytometry (Gallios, Beckman Coulter, Brea, CA, USA), and data were examined using FlowJoTM software, version 10.9 (Beckton Dickinson and Company, Franklin Lakes, NJ, USA) [34]. 

### 2.13. Statistical Analysis

SPSS statistical software version 26 (IBM, Chicago, IL, USA) and R software version 4.2.2 (The R foundation for Statistical Computing, Vienna, Austria) were utilized in this study. Data are expressed as the mean ± standard deviation (SD) of the mean. Categorical variables were examined using the chi-square test. Multi-group comparisons were conducted using an unpaired *t* test or Mann–Whitney U test. Two-Stage Hazard Rate Comparison and multivariate models with Cox proportional hazards regression analysis were used to evaluate survival. Overall survival (OS), recurrence-free survival (RFS), and disease-specific survival (DSS) were extracted from the case record as reported previously [29]. *p* < 0.05 was deemed statistically significant. 

## 3. Results

### 3.1. High FAF1 Expression Is Observed in NSCLC, but Not in Non-Tumor Lung Tissues

**Figure 1:**
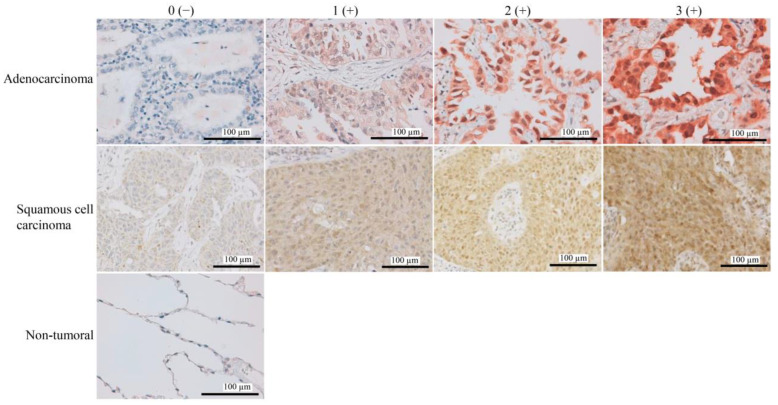
FAF1 expression in NSCLC. Representative images of various degrees of FAF1 expression in NSCLC adenocarcinoma (×40 magnification), squamous cell carcinoma (×20 magnification), and non-tumor (×40 magnification) (scale bars, 100 µm). Staining intensity was categorized as 0 (absent), 1 (weak), 2 (moderate), and 3 (strong). **Left**, **top** to **bottom**: Subtypes of adenocarcinoma based on the whole part are acinar, acinar, papillary, and lepidic.

This study included 609 NSCLC patients who had undergone surgery. The average age was 67 (range: 23–88 y). Among the patients, 415 (68.1%) were male and 416 (68.3%) had a smoking history. There were 232 (38.1%) patients who were in stage II or III. Histologically, these patients’ tumors were classified as 399 (65.5%) adenocarcinomas, 167 (27.4%) squamous cell carcinomas, and 43 (7.1%) tumors with other histologic features. To assess the protein expression of FAF1 in NSCLC, we conducted IHC analysis on tumor tissues and paired non-tumor tissues from NSCLC patients. FAF1 expression with varying degrees of brown staining in tumor tissues was localized in both the cytoplasm and nuclei of the tumoral cells, but not in non-tumor tissues. Diverse FAF1 expression levels within adenocarcinoma and squamous cell carcinoma were presented (Figure 1). This is consistent with the immunostaining manifestation of FAF1 in normal tissue from The Human Protein Atlas (https://www.proteinatlas.org/ENSG00000185104-FAF1/tissue/lung#img (accessed on 20 November 2020)). Furthermore, 408 of 609 lung cancer specimens (67.0%) had positive FAF1 expression. Among the positive FAF1 expression specimens, 226 (37.1%) were grade 1, 153 (25.1%) were grade 2, and 29 (4.8%) were grade 3. There was no statistical diversity between grades 0–3 in different pathological histology (Table S1). We then assessed whether differences in FAF1 expression were linked to clinicopathological factors in these NSCLC patients. The expression of FAF1 was statistically higher in patients with an advanced pathological stage (*p* < 0.001), an advanced extension of primary tumor size (*p* = 0.015), subsets with worse lymph node metastasis (*p* < 0.001), and patients with EGFR mutation (Figure S1) (*p* = 0.029) in the overall cases. FAF1 expression was significantly higher in advanced pathological stage adenocarcinoma (*p* = 0.002), worse regional lymph node metastasis (*p* = 0.014), and positive EGFR mutation (*p* = 0.006). FAF1 expression in squamous cell carcinoma showed no significant differences except in the advanced pathological stage (*p* = 0.029) (Table 1). 

### 3.2. High FAF1 Expression Indicates Poor Prognosis in NSCLC

**Figure 2:**
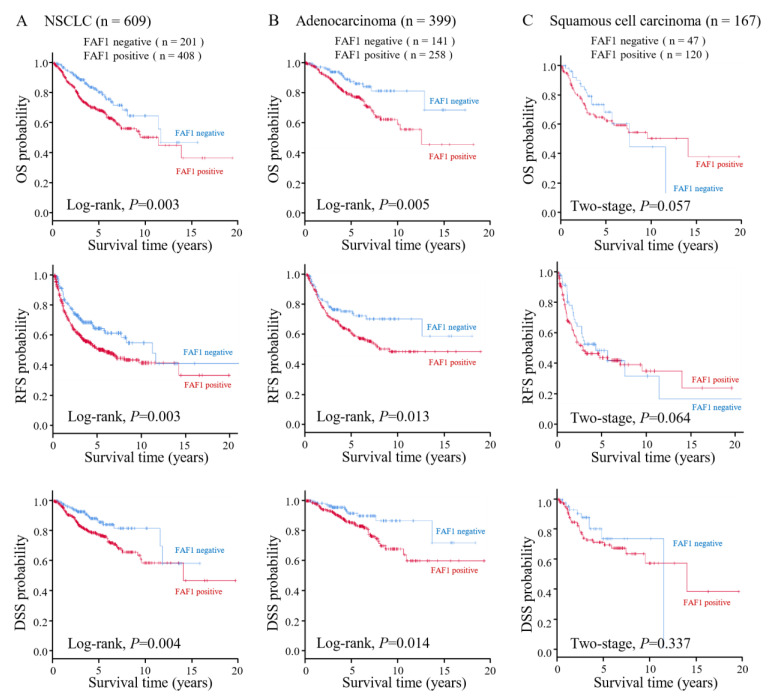
Survival analysis. Survival curves for OS, RFS, and DSS according to the expression of FAF1 in NSCLC (n = 609, log-rank test) (**A**), in adenocarcinoma (n = 399, log-rank test) (**B**), and in squamous cell carcinoma (n = 167, two-stage test) (**C**).

The prognostic value of FAF1 was assessed using Two-Stage Hazard Rate Comparison. Patients with high FAF1 expression had a significantly worse prognosis in NSCLC (log-rank *p* = 0.003 for OS, *p* = 0.003 for RFS, *p* = 0.004 for DSS) (Figure 2A). Adenocarcinoma cases had similar results (log-rank *p* = 0.005 for OS, *p* = 0.013 for RFS, *p* = 0.014 for DSS) (Figure 2B), but squamous cell carcinoma cases did not (two-stage *p* = 0.057 for OS, *p* = 0.064 for RFS, *p* = 0.337 for DSS) (Figure 2C). We also used the in silico database and the Kaplan–Meier plotter to test the prognostic association of FAF1 mRNA expression in NSCLC. As a result, in all 1411 cases, high FAF1 expression was significantly associated with poor prognosis (*p* = 0.0011), a similar result was shown in the adenocarcinoma cohort (n = 672, *p* = 0.027), but not in the squamous cell carcinoma cohort (n = 527, *p* = 0.52) (Figure S2). In silico analysis agreed with the results of the survival analysis in our cohort.

Clinicopathologic features with significant prognostic values in the two-stage test or log-rank test were used for Multivariate Cox regression analysis. According to the findings, smoking history (HR = 0.400, 95% CI = 0.194–0.824, *p* = 0.013 for adenocarcinoma), pathological stage (HR = 1.026, 95% CI = 1.004–1.049, *p* = 0.020 for all cases; HR = 1.049, 95% CI = 1.018–1.081, *p* = 0.002 for adenocarcinoma), primary tumor size (HR = 1.002, 95% CI = 1.001–1.004, *p* = 0.008 for all cases; HR = 1.003, 95% CI = 1.001–1.006, *p* = 0.006 for adenocarcinoma), and pathological histology (HR = 1.423, 95% CI = 1.113–1.820, *p* = 0.005 for all cases) were independent poor prognostic factors for OS, whereas neither positive FAF1 expression nor EGFR mutation served as statistically significant independent predictors of poor prognosis (Table 2).

### 3.3. The Relationship between FAF1 Expression and Apoptosis in NSCLC

To assess the relationship between FAF1 expression and apoptosis, TMA with 42 available NSCLC cases (38 FAF1-positive and 4 FAF1-negative) was carried out using FAF1 IHC and a TUNEL assay. The background of FAF1 staining was strong, and thus, we interpreted faint ones as negative (Figure 1), while TUNEL staining was generally weak, even in positive cases (Figure S3). As a result, apoptotic cells can be seen in NSCLC with low FAF1 expression; in contrast, few apoptotic cells were discovered in NSCLC with high FAF1 expression (*p* = 0.032) (Figure S3). This suggests that FAF1 expression is negatively associated with apoptosis in NSCLC, which is consistent with the findings of the prognostic analysis in this study. Among the 42 cases, 4 cases with EGFR mutation along with positive FAF1 expression were detected apoptosis; however, due to the limited number of the cases used in TUNEL assay, no association was found.

### 3.4. Diverse Expression of FAF1 in Lung Cancer Cell Lines 

**Figure 3:**
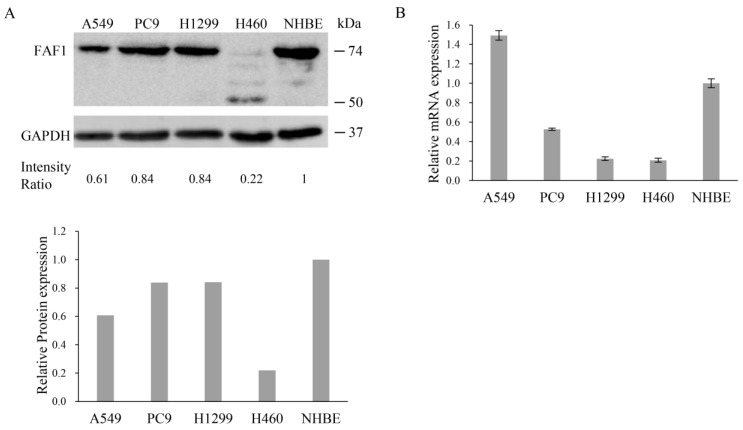
FAF1 expression in NSCLC cell lines compared with normal human bronchial epithelial cell line NHBE. (**A**) FAF1 protein expression in cell lines. Intensity Ratio of FAF1 and GAPDH was calculated and normalized via NHBE. Relative protein expression of FAF1 is shown in a bar chart. (**B**) FAF1 mRNA expression in cell lines. Each assay was performed thrice. Results are presented as means ± SD and compared to NHBE.

FAF1 expression was assessed in several lung cell lines using Western blot and qPCR. The FAF1 protein was found in the human lung cancer cell lines A549, PC9, and H1299, as well as the normal human bronchial epithelial cell line NHBE, but not in the human lung cancer cell line H460 (Figure 3A and Figure S4). A549 cells demonstrated higher mRNA expression of FAF1 than NHBE cells, as well as other human lung cancer cells (PC9, H1299, and H460) (Figure 3B). To investigate the impact of FAF1 downregulation, the cell line H460 was selected to transfect the FAF1 expression plasmid based on the result of Western blotting, and additionally, the cell line A549 was selected to perform FAF1 knockdown using siRNA based on the result of qPCR.

### 3.5. FAF1 Downregulation Inhibits the Growth of the Lung Cancer Cell Line A549 

**Figure 4:**
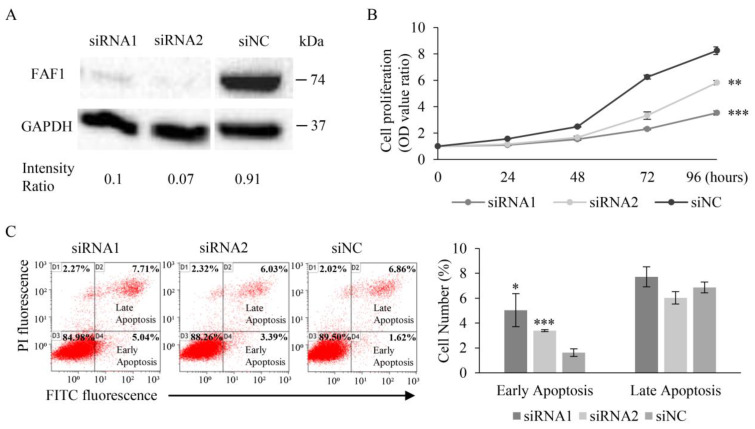
The effect of FAF1 downregulation in A549 cells. (**A**) Effective siRNA mediated decreased FAF1 expression in A549 cells. Intensity Ratio of FAF1 and GAPDH was calculated. (**B**) Effect of decreased FAF1 on cell proliferation in A549 cells. (**C**) Changes in apoptotic cell number after transfecting with FAF1 siRNA. Late-apoptosis cells are shown in the upper right quadrant, and early-apoptosis cells are shown in the bottom right quadrant of the representative scatter plot. The percentage of apoptotic cells is shown in a bar chart. Each assay was performed thrice. Results are presented as mean ± SD and compared to cells transfected with negative control siRNA (siNC); * *p* < 0.05, ** *p* < 0.01, *** *p* < 0.001 according to unpaired *t* test analysis.

To examine the effects of FAF1 on apoptosis, the human lung adenocarcinoma cell line A549 was used for the knockdown of FAF1 mRNA. A549 has wild-type TP53 and EGFR, as well as mutant-type KRAS; no specific changes in FAF1 were recorded in A549 according to the Catalog of Somatic Mutations in Cancer (COSMIC). After knocking down FAF1 with siRNA, FAF1 expression declined in A549 cells, compared to A549 cells with negative control siRNA (siNC) (Figure 4A and Figure S5). As depicted in Figure 4B, cell proliferation was significantly reduced by the downregulation of FAF1. In view of the fact that FAF1 acting upstream of caspase-8, which is a member of initiator caspases and play a role of an apoptosis activator [6,35,36], we discriminated the early and late stages of apoptosis in flow cytometry analysis. Flow cytometry analysis revealed that the downregulation of FAF1 significantly increased the number of A549 cells (siRNA1, 5.04%, *p* = 0.01; siRNA2, 3.39%, *p* < 0.001) in the early stages of apoptosis when compared to the negative control (siNC, 1.62%) (Figure 4C). However, the ‘promotion of early apoptosis’ cannot be concluded despite the apparent statistical significance due to the max rate of apoptosis cells being <10%. These findings indicate that FAF1 downregulation inhibits cell growth and may promote early apoptosis in the NSCLC cell line A549, which is consistent with the findings of the prognostic analysis and TUNEL assay in this study.

### 3.6. FAF1 Overexpression Has No Effect on the Growth of the Lung Cancer Cell Line H460

**Figure 5:**
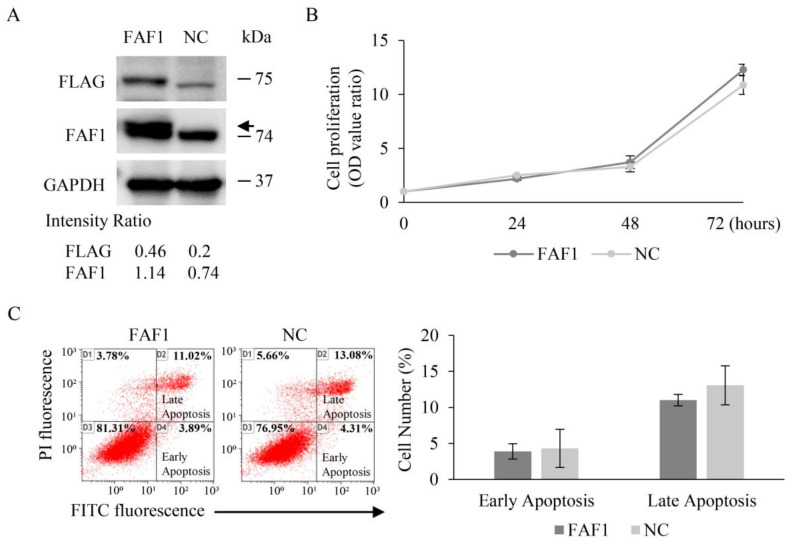
The effect of FAF1 overexpression in H460 cells. (**A**) Effective FAF1 expression plasmid transfection in H460 cells. The arrow represents FAF1 with a Flag tag (75 kDa). Intensity Ratios of FLAG and GAPDH, and FAF1 and GAPDH, were calculated. (**B**) No effect was shown on cell proliferation in H460 cells after being transfected with the FAF1 expression plasmid. (**C**) The number of apoptotic H460 cells after transfecting with the plasmid vector. Late-apoptosis cells are shown in the upper right quadrant, and early-apoptosis cells are shown in the bottom right quadrant of the representative scatter plot. The apoptosis rates in cells with vector transfection are shown in a bar chart. Each assay was performed thrice. Results are presented as mean ± SD and compared to the negative control (NC: H460 cells transfected with empty vector).

To examine the effects of apoptosis due to FAF1 overexpression, we chose H460 to transfect the FAF1 plasmid because it has low FAF1 expression. The human large-cell lung cancer cell line H460 also has wild-type TP53 and EGFR, as well as mutant-type KRAS. No specific changes in FAF1 were recorded in the COSMIC cell line database. Endogenous FAF1 expression was greatly increased in H460 cells due to stimulation with vector transfection. Cells transfected with a FAF1 expression plasmid also had a slightly larger-sized Flag-FAF1 proteins than the negative control that cells transfected with an empty vector (Figure 5A and Figure S6). Transfection efficiency of 40.8% was confirmed via immunofluorescence staining (Figure S7). Neither cell proliferation nor cell apoptosis changed considerably after transfecting the FAF1 expression plasmid compared to the negative control, although the degree of FAF1 expression varied (Figure 5B,C). Actually, FAF1 expression increased even in H460 with transfection of the empty plasmid, compared to the absent expression shown in H460 (Figure S8). We supposed that endogenous FAF1 was probably induced by the transfection procedure itself. The mechanistic basis of this phenomenon is unknown, but in any case, we could not obtain a significant difference in apoptosis in H460 transfected with the FAF1 expression vector.

## 4. Discussion

In this study, we discovered that FAF1 was significantly higher in tumoral tissues compared to non-tumoral tissues in NSCLC, and that it was significantly associated with poor prognosis, particularly in adenocarcinoma. Furthermore, the downregulation of FAF1 significantly inhibited cell viability and tended to promote early apoptosis in a lung adenocarcinoma cell line. Our findings suggest that FAF1 plays a variety of roles in NSCLC progression, in addition to acting as a tumor suppressor.

Previous research found that functional loss, rather than the overexpression of FAF1, was frequently found in cancers of various origins. In fact, FAF1 expression was found to be downregulated in a significant proportion of human gastric carcinomas [17,18]. Furthermore, nearly 30% of uterine cervix carcinomas have a genomic loss at the FAF1 locus [15]. Recurrent mono-allelic and homozygous deletion of FAF1 has been discovered in mantle cell lymphoma [16]. Furthermore, single-nucleotide polymorphism testing revealed that FAF1 may be linked to a genetic locus implicated in susceptibility to Crohn’s disease, an inflammatory bowel disorder associated with an increased risk of colorectal cancer [21]. Recently, germline FAF1 mutations have been discovered in familial colorectal cancer (CRC), and these variants encode an unstable form of FAF1 that increases the resistance of CRC cells to apoptosis [22]. Clearly, these studies have classified FAF1 as a tumor suppressor. However, the manifestation found here demonstrated that, contrary to previous reports, FAF1 reverses its role as a tumor suppressor for unknown reasons in NSCLC. 

There is a lack of data on FAF1 in lung cancer. Wei et al. discovered that FAF1 RNA expression in NSCLC was lower than in non-tumor tissue, and that sanguinarine inhibits tumor growth by upregulating FAF1 in NSCLC [19]. However, FAF1 protein expression in NSCLC tissue was not discussed in their paper. Ye et al., on the other hand, identified miR-26a-5p as an oncogenic miRNA that influences NSCLC progression, and proposed that this phenomenon is caused by the downregulation of FAF1 by miR-26a-5p [37]. They did not show FAF1 expression in NSCLC tissue either. Both studies suggest FAF1 as a tumor suppressor in NSCLC, which contradicts our findings. In our study, FAF1 protein expression was semi-quantified using IHC analysis, which has never been achieved before. We were unable to quantify RNA expression in NSCLC tissues in our own cohort due to a lack of tissue. Nonetheless, as we can see here, The Cancer Genome Atlas dataset suggests that the upregulation of FAF1 RNA is associated with poor prognosis in NSCLC, when analyzed using the Kaplan–Meier plotter. We do not know the precise reason why the previous two studies using proteomics and RNA produced inconsistent results with ours and the TCGA dataset.

The protein expression patterns of FAF1 were different from the mRNA expression patterns of FAF1 among the cell lines detected in this study. Considering that it may be FAF1 gene alteration that affects protein expression, we searched COSMIC, and no FAF1 mutation was recorded in these cell lines. Imperfect correlations between mRNA and protein were thought to arise due to technical (for example, measurement errors) or biological (for example, post- transcriptional regulation) reasons. Gene expression is a complex process that is regulated on many levels. Moreover, protein translation is itself a complex multistep process that is subject to extensive regulation at the levels of initiation, elongation, localization, and ribosome composition [38,39]. NHBE may not be suitable for function experiments, because FAF1 expression was detected in NHBE but not in clinical non-tumoral tissues in this study, so utterly different environments to those in which cells live were considered [40]. Moreover, H460 cells barely expressed FAF1 at 74 kDa, and they seemed to expressed possible proteins of a lower molecular weight of 50 kDa, as shown in Figure 3A. FAF1 encodes a 74 kDa protein as well as a 57 kDa protein through alternative splicing. We supposed that the protein that appeared in H460 cells could be a 57 kDa protein. Compared to the 74 kDa protein, aa188–399 in the 57 kDa protein is deleted, which contains the DEDID or UBL2 domain. The interaction of the FAF1-DEDID with the death effector domains (DEDs) of FADD and caspase-8 resulted in enhanced death effector filament (DEF) formation and apoptosis. The UBA domain is crucial for FAF1-mediated apoptosis and proteasomal inhibition; however, the FAF1 UBA domain dose not directly interact with the UBL1 and UBL2 or UBX domains [8,9,41]. Thus, we supposed that the deletion of DEDID or UBL2 could weaken DEF formation and apoptosis, or have no effect on apoptosis and proteasomal inhibition. The function of the 57 kDa protein has barely been reported; it will be interesting to investigate it. Intriguingly, FAF1 (74 kDa) was detected when H460 cells were transfected with an empty vector, the negative control in Figure 5A and Figure S8. It is possible that damage to the cells during the transfection of the plasmid vector may increase endogenous FAF1 expression by increasing FAF1 mRNA expression or inhibiting FAF1 protein degradation.

In this study, FAF1 was detected in both the nucleus and cytoplasm of NSCLC tissue. Previous studies showed FAF1 enhances apoptosis by interacting with Fas in the cytoplasm; on the other hand, it modulates a variety of biological processes by interacting with diverse molecules in the nucleus [5,9]. The function of FAF1 correlation with FAS was not studied in this paper, due to the phenomenon that FAF1 showed high expression in the nucleus but failed to enhance apoptosis. We hypothesize that FAF1 in the nucleus plays a dominant role in NSCLC. In our cohort, the expression of FAF1 was statistically higher in patients with an advanced pathological stage, an advanced extension of primary tumor size, and subsets with worse lymph node metastasis, respectively. At the same time, high FAF1 expression also showed a worse prognosis. We thought that it furthermore illustrates that FAF1 in NSCLC does not act as a tumor suppressor. These results are not logically contradictory to the conclusion that better prognosis occurs in most patients with early-stage NSCLC. Besides the advantage of treatment in early-stage NSCLC [42], we speculate that FAF1 may participate in the self-repair of cells in the early stage, which remains to be study. NSCLC specimens with an EGFR mutation (the exon19 deletions or the L858R mutation in exon 21) had significantly higher FAF1 expression, and apoptotic cells were observed. We do not know why these associations occurred in lung cancer, and no information about the interaction of FAF1 and EGFR has been reported thus far. This observation could be explained by a single candidate molecule. XIAP inhibits FAF1-mediated cell death by interfering with the caspase cascade and directly interferes with the NF-κB pathway [43], and XIAP deficiency resulted in the remarkable suppression of EGFR expression [44]. The interaction of XIAP, FAF1, and EGFR would be an interesting area to explore in lung carcinogenesis. However, FAF1 participates in a variety of cell pathways, and a complete understanding of FAF1’s role in NSCLC is still a long way off. 

In our cohort, multivariate Cox regression analysis did not reveal that FAF1 is an independent prognostic factor, implying that FAF1 is not involved in the biological malignancy of NSCLC. There are several possible explanations to consider. Although EGFR mutation is widely recognized as a driving event in NSCLC, it showed no statistical significance in this study, indicating that more specimens should be used. In this study, FAF1 downregulation in the lung adenocarcinoma cell line A549 promoted early apoptosis, whereas no conclusion could be reached when FAF1 was upregulated in the lung large-cell carcinoma cell line H460. Therefore, the role of FAF1 in NSCLC cannot be easily summarized. Above all, FAF1 can be regarded as a risk factor in NSCLC, rather than an independent influence factor. 

This research has some limitations. We semi-quantified FAF1 expression at the protein level in NSCLC tumor and non-tumor tissue without measuring it at the RNA level. Therefore, we could not rule out the possibility that changes in protein translation factors cause high FAF1 protein expression, or that RNA instability causes FAF1 retention. We only studied one cell line with up- and downregulated FAF1. Thus, in vitro recapitulation of the tumorigenic role of FAF1 may be insufficient when considering a variety of lung cancer cells, for example, with or without EGFR mutation. We only tried lipofectamine to transfect the FAF1 plasmid into the cell line, although other methods like viral transfection can be utilized. We removed dead cells in the medium before cells were collected for apoptosis analysis, which made the total apoptosis cells occupy a low proportion. We detected only classical EGFR mutations associated with increased sensitivity to EGFR tyrosine kinase inhibitors (TKI). TKI resistance mutations, such as T790M, are also important when studying treatments. We should perform TUNEL assay on more cases with EGFR mutation. We did not conduct additional research to determine the relationship between EGFR mutation or NF-κB activation and FAF1 expression in NSCLC. It is preferable to perform RNA Seq analysis and cell line transplant experiments, and we will consider this in a further study. 

The results in this paper show that FAF1 needs further study to figure out the complex mechanism through which it works in the apoptosis process. FAF1 is still a candidate target for anti-cancer therapy development. Recently, studies have shown that the bioactive ingredients of natural products like hesperidin affect various cancers by modulating the various cell signaling pathways, which include apoptosis and the cell cycle et al. [45]. Hesperidin may work well in NSCLC with high FAF1 expression. We would like to confirm this in a future study.

## 5. Conclusions

Our findings show that FAF1 is overexpressed in NSCLC, which is associated with a poor prognosis. It appears that FAF1 has a variety of bio-functions in NSCLC in comparison to the presumption that it enhances or initiates apoptosis.

## Figures and Tables

**Table 1:** Characteristics of NSCLC according to FAF1 expression.

	All Cases	FAF1 in Nuclei			Ad	FAF1 in Nuclei			Sq	FAF1 in Nuclei		
Factor	Total *n* = 609	Negative *n* = 201 (33%)	Positive *n* = 408 (67%)	*p*-Value	Total *n* = 399	Negative *n* = 141 (35.3%)	Positive *n* = 258 (64.7%)	*p*-Value	Total *n* = 167	Negative *n* = 47 (28.1%)	Positive *n* = 120 (71.9%)	*p*-Value
Mean Age (range)	67 (23~88)				66 (23~86)				70 (39~88)			
<Mean Age	248 (40.7)	78 (31.4)	170 (68.6)	0.499	179 (44.9)	62 (34.6)	117 (65.4)	0.791	83 (49.7)	21 (25.3)	62 (74.7)	0.417
≥Mean Age	361 (59.3)	123 (34.1)	238 (65.9)		220 (55.1)	79 (35.9)	141 (64.1)		84 (50.3)	26 (31)	58 (69)	
Gender												
Female	194 (31.9)	69 (35.6)	125 (64.4)	0.358	178 (44.6)	65 (36.5)	113 (63.5)	0.659	156 (93.4)	44 (28.2)	112 (71.8)	0.947
Male	415 (68.1)	132 (31.8)	283 (68.2)		221 (55.4)	76 (34.4)	145 (65.6)		11 (6.6)	3 (27.3)	8 (72.7)	
Smoking History												
Never	181 (29.7)	63 (34.8)	118 (65.2)	0.656	174 (43.6)	62 (35.6)	112 (64.4)	0.928	5 (3.0)	1 (20)	4 (80)	0.666
Ever	416 (68.3)	137 (32.9)	279 (67.1)		219 (54.9)	79 (36.1)	140 (63.9)		156 (93.4)	45 (28.8)	111 (71.2)	
Unknown	12 (2.0)	1	11		6 (1.5)	0	6		6 (3.6)	1	5	
Pathological Stage												
I	377 (61.9)	148 (39.3)	229 (60.7)	<0.001	276 (69.2)	111 (40.2)	165 (59.8)	0.002	84 (50.3)	30 (35.7)	54 (64.3)	0.029
II, III	232 (38.1)	53 (22.8)	179 (77.2)		123 (30.8)	30 (24.4)	93 (75.6)		83 (49.7)	17 (20.5)	66 (79.5)	
Primary Tumor												
T1	246 (40.4)	95 (38.6)	151 (61.4)	0.015	199 (49.9)	75 (37.7)	124 (62.3)	0.327	38 (22.8)	15 (39.5)	23 (60.5)	0.077
T2,3,4	363 (59.6)	106 (29.2)	257 (70.8)		200 (50.1)	66 (33.0)	134 (67.0)		129 (77.2)	32 (24.8)	97 (75.2)	
Lymph Nodes Met												
N0	452 (74.2)	168 (37.2)	284 (62.8)	<0.001	312 (78.2)	120 (38.5)	192 (61.5)	0.014	117 (70.1)	38 (32.5)	79 (67.5)	0.057
N1,2,3	157 (25.8)	33 (21.0)	124 (79.0)		87 (21.8)	21 (24.1)	66 (75.9)		50 (29.9)	9 (18.0)	41 (82.0)	
Pathological Histology												
Ad	399 (65.5)	141 (35.3)	258 (64.7)	0.233								
Sq	167 (27.4)	47 (28.1)	120 (71.9)									
Others	43 (7.1)	13 (30.2)	30 (69.8)									
EGFR Mutation												
Negative	484 (79.5)	170 (35.1)	314 (64.9)	0.029	286 (71.7)	113 (39.5)	173 (60.5)	0.006	159 (95.2)	44 (27.7)	115 (72.3)	0.546
Positive	125 (20.5)	31 (24.8)	94 (75.2)		113 (28.3)	28 (24.8)	85 (75.2)		8 (4.8)	3 (37.5)	5 (62.5)	

Abbreviations: Ad, adenocarcinoma; Sq, squamous cell carcinoma; Met, metastasis.

**Table 2:** Multivariate Cox hazard models of overall survival in NSCLC.

	All Cases			Ad			Sq		
Factor	HR	95.0% CI	*p*-Value	HR	95.0% CI	*p*-Value	HR	95.0% CI	*p*-Value
Gender (female, male)	0.791	0.466–1.344	0.387	1.253	0.655–2.398	0.495	0.792	0.210–2.987	0.731
Smoking History (never, ever)	0.679	0.366–1.259	0.219	0.400	0.194–0.824	0.013	2.338	0.485–11.275	0.290
Pathological Stage (I, II, III)	1.026	1.004–1.049	0.020	1.049	1.018–1.081	0.002	1.004	0.967–1.042	0.841
Primary Tumor (T1, T234)	1.002	1.001–1.004	0.008	1.003	1.001–1.006	0.006	1.001	0.998–1.004	0.446
Regional Lymph Nodes (N0, N123)	1.311	0.826–2.081	0.251	0.912	0.487–1.707	0.772	1.960	0.870–4.413	0.104
Pathological Histology (Ad, Sq, others)	1.423	1.113–1.820	0.005						
EGFR Mutation (negative, positive)	0.940	0.589–1.502	0.797	0.963	0.547–1.695	0.895	0.530	0.115–2.451	0.417
FAF1 in Nuclei (negative, positive)	1.313	0.901–1.912	0.156	1.709	0.984–2.968	0.057	1.002	0.545–1.840	0.996

Abbreviations: HR, hazard ratio; CI, confidence interval; Ad, adenocarcinoma; Sq, squamous cell carcinoma.

## Data Availability

The data presented in this study are available on request from the corresponding author. The data are not publicly available due to privacy reasons.

## Supplementary Materials

The following supporting information can be downloaded at: https://www.mdpi.com/article/10.3390/curroncol30110687/s1, Table S1: Categorical data of FAF1 expression grade; Figure S1: IHC images of EGFR mutants; Figure S2: Prognostic association of FAF1 mRNA expression in NSCLC shown in Kaplan–Meier plotter; Figure S3: TUNEL-positive cells occurred in NSCLC with FAF1-negative expression; Figure S4: Original data of Figure 3A; Figure S5: Original data of Figure 4A; Figure S6: Original data of Figure 5A; Figure S7: Immunofluorescence detection of FAF1 protein in H460 cells; Figure S8: FAF1 expression in H460 cells.
