# Supplementary material for: High Expression of Fas-Associated Factor 1 Indicates a Poor Prognosis in Non-Small-Cell Lung Cancer
Source: Curr Oncol. 2023 Oct 25;30(11):9484–500. doi: 10.3390/curroncol30110687 (PMC10670600; doi:10.3390/curroncol30110687)
Supplement: Supplementary file 1 [file curroncol-30-00687-s001.zip › curroncol-2648239-supplementary.pdf]

Table S1. Categorical data of FAF1 expression grade.

	FAF1 expression grade in nuclear					
	**n (%)**	**0**	**1**	**2**	**3**	***p*-value**
**All**	609	201 (33)	226 (37.1)	153 (25.1)	29 (4.8)	
**Adenocarcinoma**	399 (65.5)	141 (35.3)	142 (35.6)	101 (25.3)	15 (3.8)	0.326
**Squamous cell carcinoma**	167 (27.4)	47 (28.1)	70 (41.9)	40 (24.0)	10 (6.0)	
**Others**	43 (7.1)	13 (30.2)	14 (32.6)	12 (27.9)	4 (9.3)	

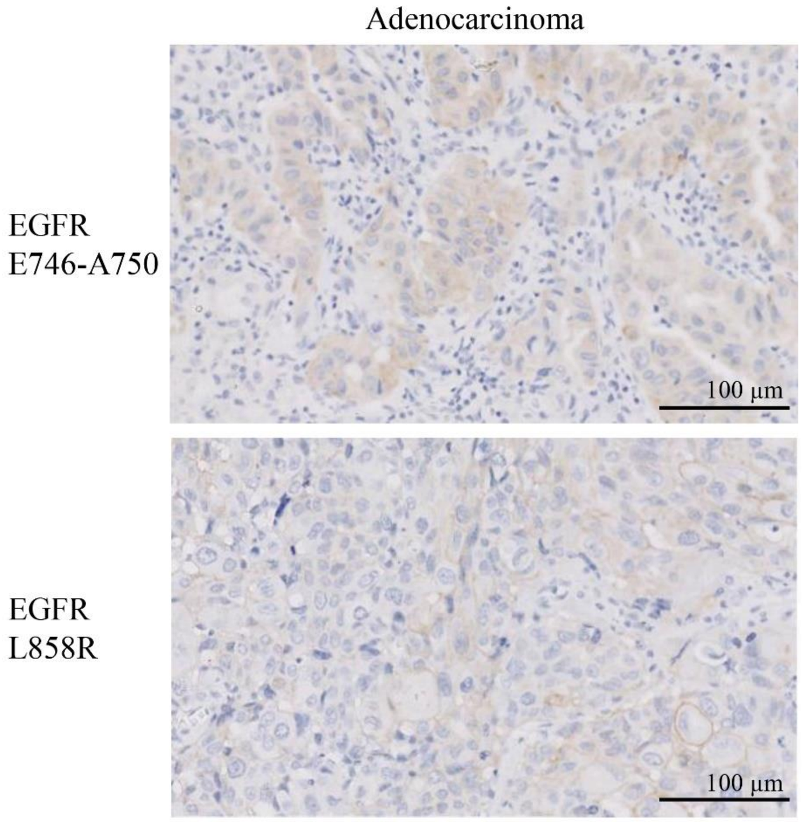

Figure S1. IHC images of EGFR mutants. Upper panel: EGFR E746-A750 in acinar adenocarcinoma of lung.

Lower panel: EGFR L858R in acinar adenocarcinoma of lung. (×20 magnification; scale bars, 100μm)

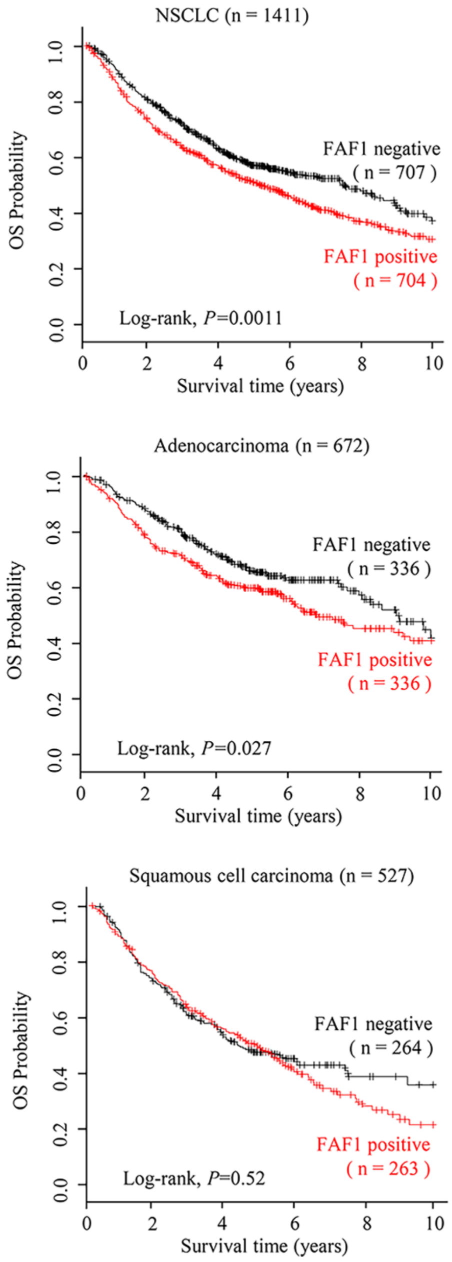

Figure S2. Prognostic association of *FAF1* mRNA expression in NSCLC showed in Kaplan-Meier plotter. Tumors with high *FAF1* expression were significantly associated with poor prognostic except squamous cell carcinoma (NSCLC, p = 0.0011; Ad, p = 0.027; Sq, p = 0.52).

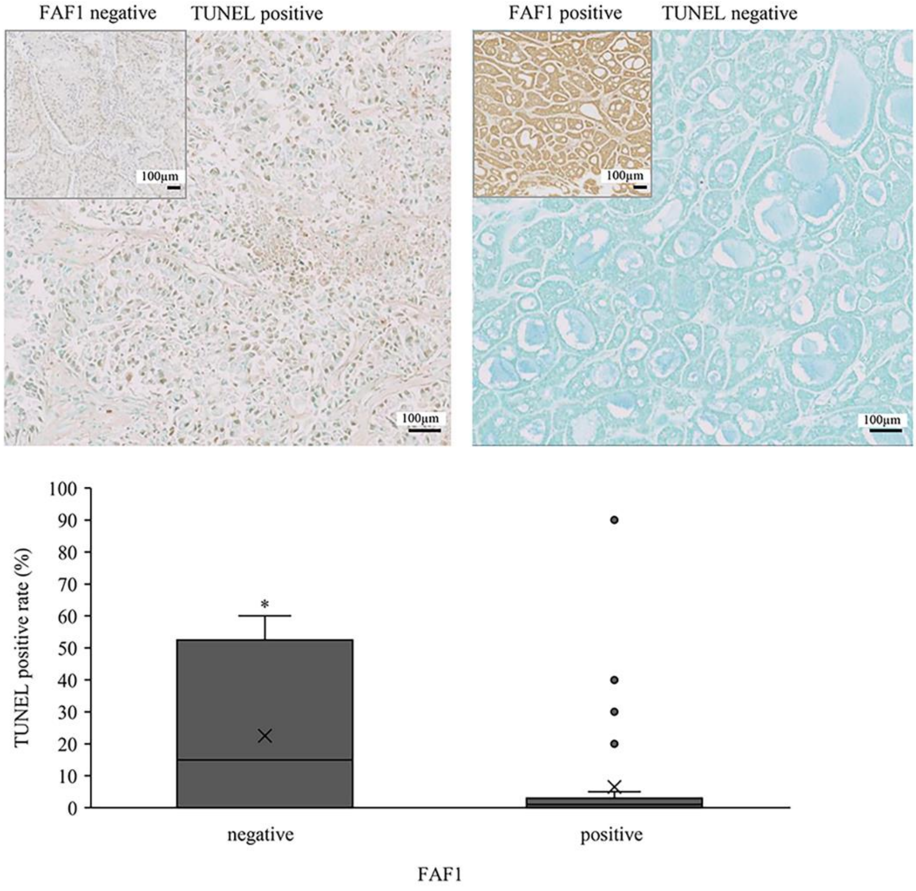

Figure S3. TUNEL positive cells occurred in NSCLC with FAF1 negative expression. Left: FAF1 negative, TUNEL positive case. Right: FAF1 positive, TUNEL negative case. (×10 magnification; scale bars, 100µm). Below: TUNEL positive cells (n=2) in FAF1 negative cases (n=4) were significantly more abundant than TUNEL positive cells (n=4) in FAF1 positive cases (n=38) (*p = 0.032, Mann-Whitney U test). The cross indicated the mean value, the upper horizontal bar indicated the maximum value, the inner horizontal bar indicated the median value, and points in the FAF1 positive bar chart indicated *EGFR* mutation cases. Subtype of adenocarcinoma based on the whole part is, acinar (left), bronchogenic (right).

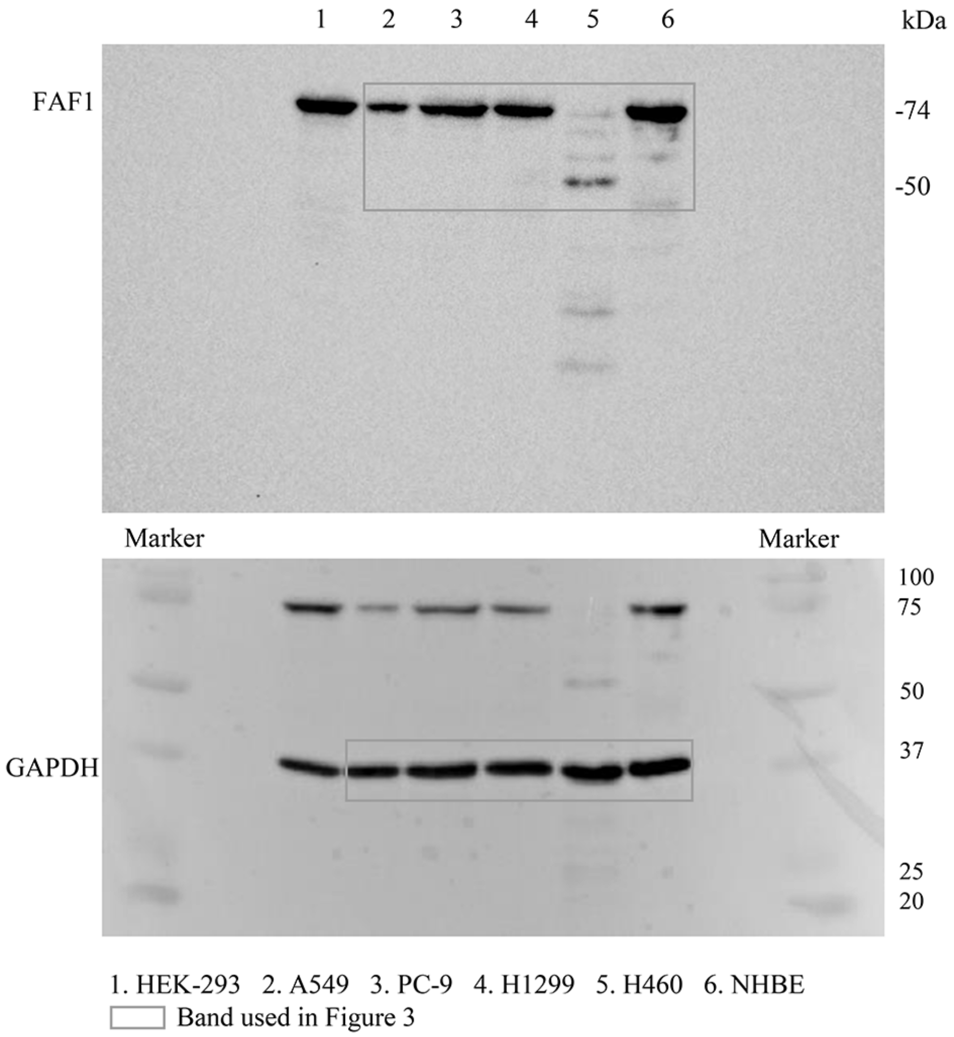

Figure S4. Original blot of Figure 3A. FAF1 protein expression in cell lines.

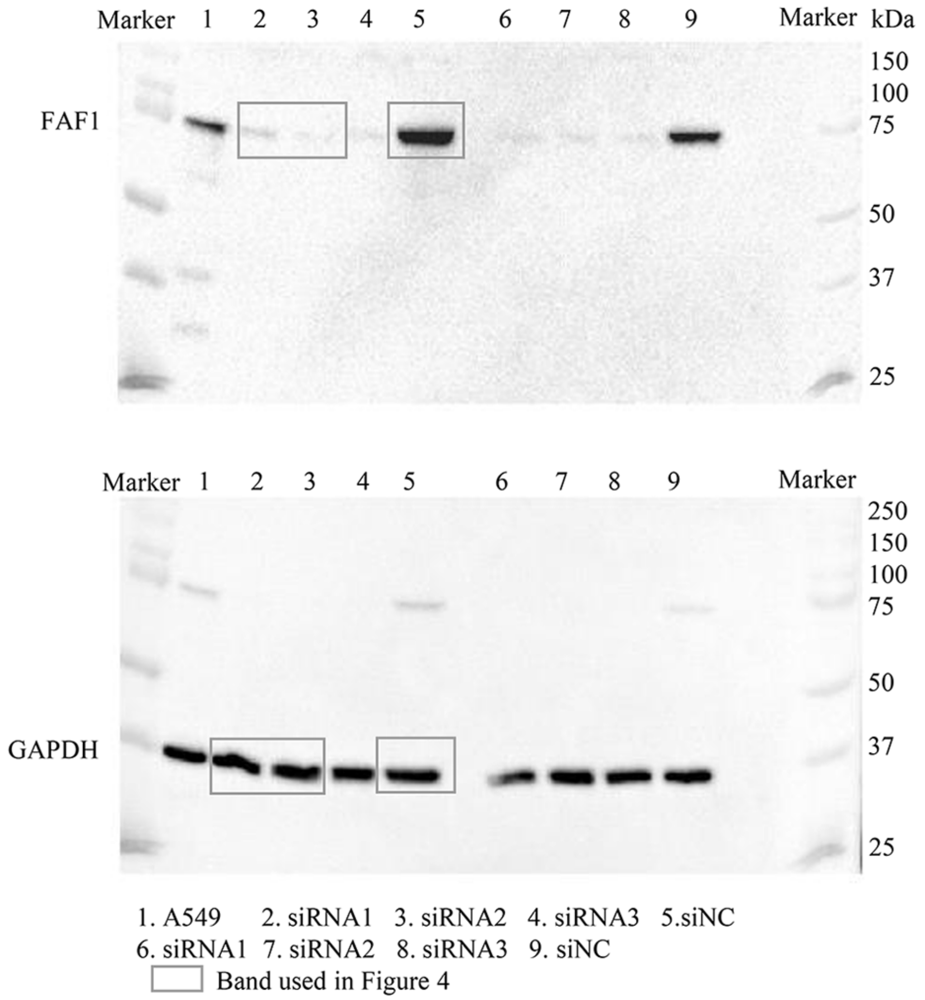

Figure S5. Original blot of Figure 4A. Effective siRNA mediated decreased FAF1 expression in A549 cells.

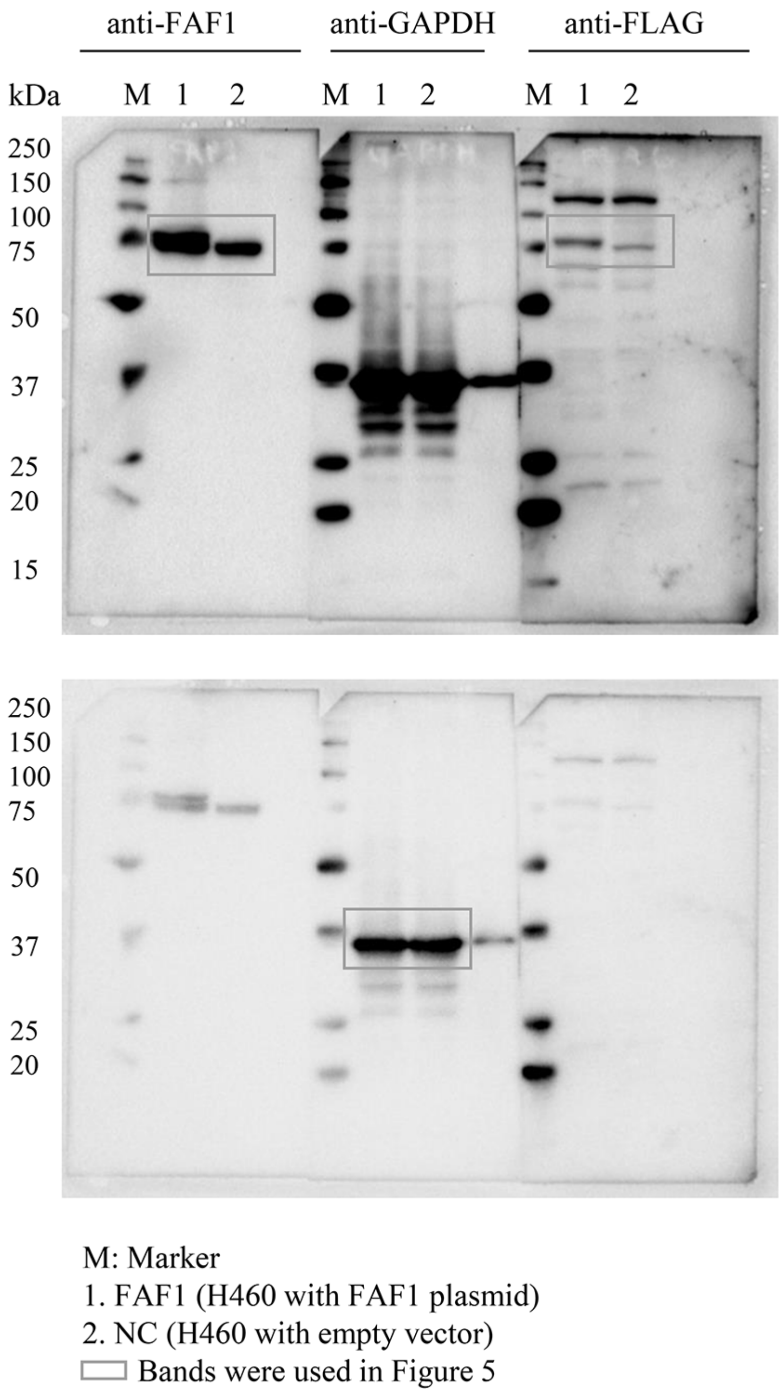

Figure S6. Original blot of Figure 5A. Effective FAF1 expression plasmid transfection in H460 cells. The same blot under different exposure time was shown to make sure band of GAPDH appear clearly (upper and lower).

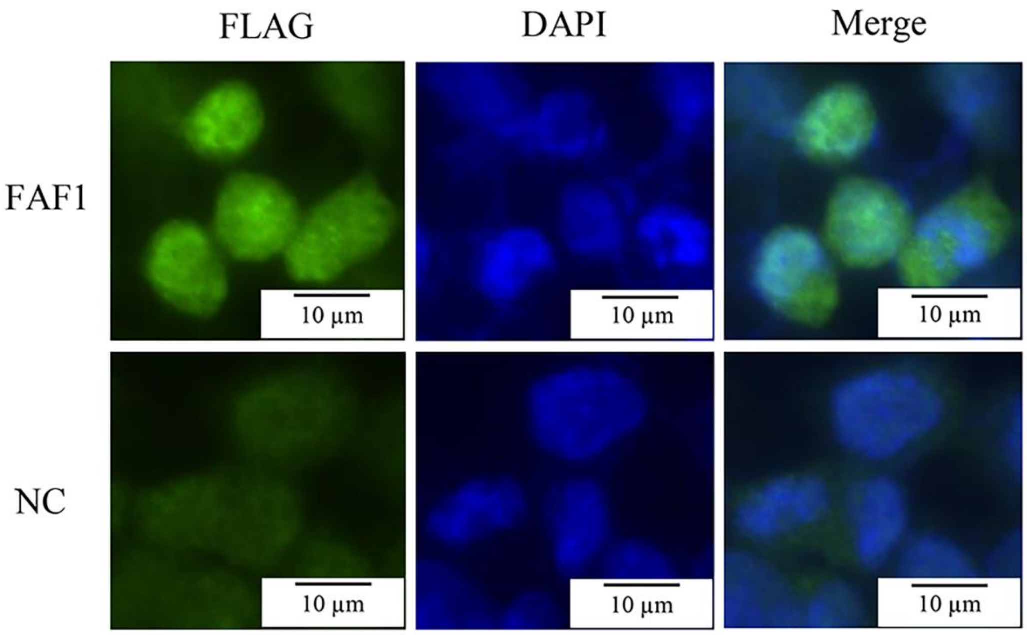

Figure S7. Immunofluorescence detection of FAF1 protein in H460 cells (×40 magnification; scale bars, 10μm). FAF1 protein (green) was stained with an anti-Flag primary antibody and an Alexa Fluor 488 goat anti-rabbit IgG secondary antibody. Nuclei were counterstained with 4′,6′-diamidino-2-phenylindole (blue).

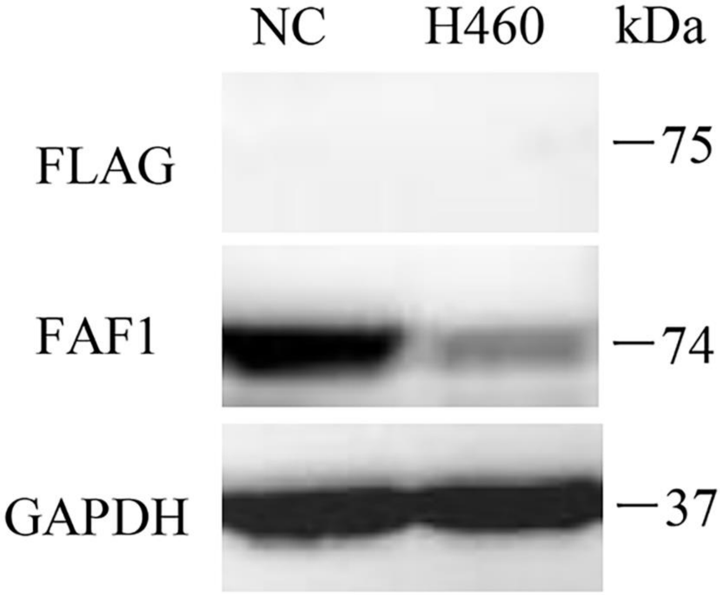

Figure S8. FAF1 expression in H460 cells. Absent FAF1 expression in H460 cells with no transfection, compare with H460 cells transfected with empty vector (NC).
